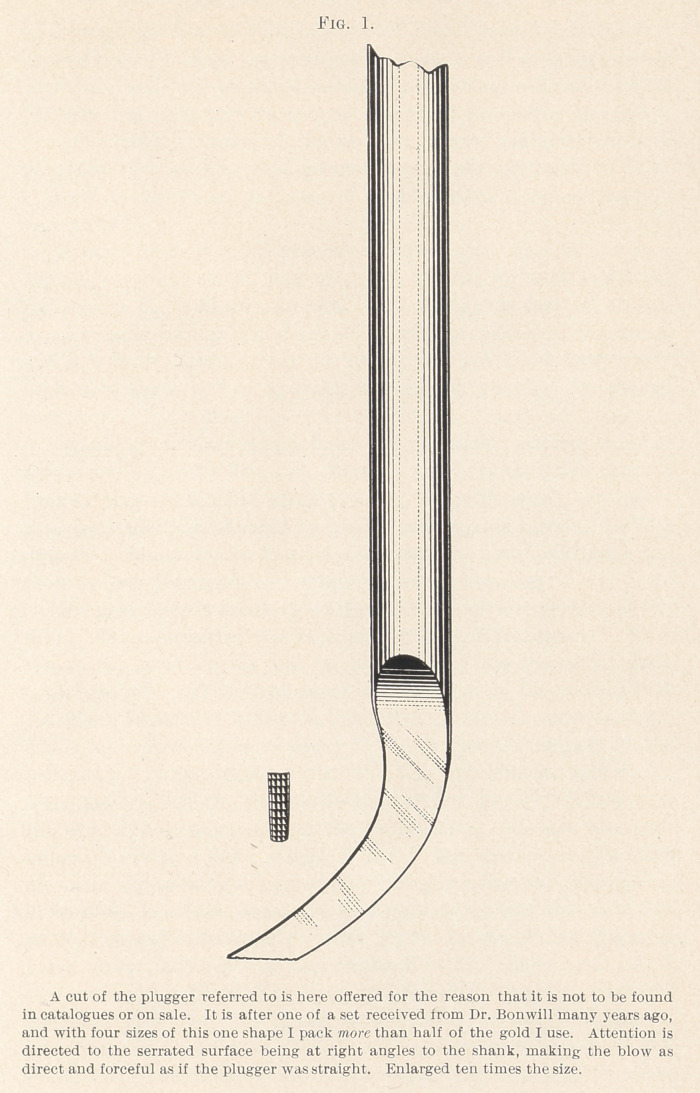# Dr. Meyer L. Rhein’s Technique

**Published:** 1904-07

**Authors:** Charles Southwell

**Affiliations:** Milwaukee, Wis.


### DR. MEYER L. RHEIN'S TECHNIQUE.

BY DR. CHARLES SOUTHWELL, MILWAUKEE, WIS.

Dickens, in speaking of Daniel Doyce, said, "Showed the whole thing as if the Divine artificer had made it and he happened to find it. So modest was he about it, such a pleasant touch of respect mingled with his quiet admiration of it and so calmly convinced that it was established on irrefragible laws."

Dr. Meyer L. Rhein, of New York City, read a paper entitled "The Technique of Approximal Restorations with Gold in Posterior Teeth," before the Academy of Stomatology, in Philadelphia, in February, 1903. The paper appears in the International Dental Journal, September, 1903, in which number may also be found an extended discussion at page 711. A *résumé* by Dr. Wedelstaedt may be found on page 25 in the January, 1904, number of the same journal. Dr. Rhein's reply to Dr. Wedelstaedt may be found in the March, 1904, number.

When an essayist appears before the Academy of Stomatology of Philadelphia, a body of gentlemen composed of professors, clinical teachers, and accredited experts, it is to be presumed that his effort will approach the scientific, and that any new data or expedient offered the subject under consideration will have sufficient dignity to warrant an introduction, and furthermore that the diction be clear in construction and chosen with a distinct desire to be instructive and deferential in view of the attainments of his audience.

To one critically inclined a close examination of Dr. Rhein's paper reveals marked ability in the construction of a very presentable garment out of old material and a distinct effort of the writer to interject inconsequent somethings, all of which when published in dental journals pictures Dr. Rhein as a sizar of operative dentistry, and places before the inexperienced teachings that are unavailing if not mischievous. Much if not all of the commendable matter in his paper may be safely said to have been covered by others in the past.

Taking up the components of his paper *seriatim,* attention is directed to the title, The Technique, etc. He might have said "a history of," or "some suggestions in," or "a later-day consideration of," and one may be excused in noting the modesty.

On page 654 he directs that the cervical wall (meaning foundation or floor) be "made perfectly flat, in order to form a stable foundation for the filling." Dr. Rhein's plethora of the theoretical compelled him to say perfectly flat when he meant reasonably flat. He immediately follows this with the statement that any deviation from such an ideal lessens the ability of the filling to withstand the strain of usage. Viewed from a practical standpoint this is theoretical rot, in the estimation of the writer, for the suggested change from a "natural rounded outline of the cervical margin, especially at the angles," is obviously for extension only. Certainly not for stability, for it should be obvious that a V-shaped filling, broad or wide, and with bevelled margins at the masticating surface, may end in an edge at the cervical margin at the centre of the interspace, and be as enduring, so far as stress is concerned, as any other filling, if usual undercuts and good condensation be had. If it fails, it will be from a recurrence of decay on one or both sides of the cervical end of the V. This clearly points to extension for prevention only. Should a filling of the character referred to by Dr. Rhein need a flat floor for stability (and it is not admitted), that requirement may in part be better provided by bevelling the coronal or masticating ends of the walls, a procedure (mentioned by Dr. Rhein) that has been audited for many years.

He argues against undercuts, and, referring to Fig. 1, states that "the real anchorage seat of all fillings of this nature depends on the dovetailed occlusal step cut at right angles into the occlusal surface," following this with directions for the cutting of a noncarious occlusal surface. Can any exigency in the preparation of approximal cavities justify the mutilation of a non-carious fissure in a bicuspid to the degree necessary to form "the real anchorage"? And yet he clearly directs this to be done. If the fissure be not involved, the walls will accept a liberal undercut. If the fissures be involved, the step may be relied on for anchorage, but as no such distinction is made in his paper, it is reasonable to assume that he dispenses with required undercuts in the cavity proper, and, depending largely on the step, mutilates all innocent fissures. In a bicuspid this is malpractice, and in a molar inexcusable.

He decries undercuts, but advises dovetailing. All very pretty on paper or in pine, but so far as the weakening of the walls is concerned, wherein lies the difference? As to the forming of undercuts and dovetailing, the former may be undulating, following the lines outlined by caries, and are quickly made with a bur, while the latter dictates a straight wall in which the dovetail is laboriously and painfully made with edge tools.

It should be obvious to any one of extended experience that undercuts need not be deep enough to weaken the walls. Moderate undercuts, but little more than sufficient to keep the filling from rocking during the packing of the foil, will retain a perfectly packed filling of foil if mainly cohesive.

Passing some commendable matter, in which he directs the packing of the gold, we arrive at the injunction to wash the cavity "with a few drops of ten per cent. solution of formalin and then thoroughly dry." How profound! Does he imagine that any one in his audience or elsewhere, deciding that formalin be advisable, would use a teaspoonful or a hose? Dry it thoroughly, he says, quite as a kindergartner might say, after the tots are ready to be sent home, "Now, children, do not get your feet wet."

It is next directed that a small piece of one of the plastic forms of gold be placed on the electric annealer.

A small amount of oxyphosphate is now mixed to the consistency of thick cream, and an amount equal to the head of a pin is placed carefully *along* the inner half of the floor, being careful not to allow any of this small amount to remain on the margins. This caution to that audience!

The "very small piece" of plastic gold which has flocked all alone on the electric annealer, at Dr. Rhein's direction, is now to be remembered and "carefully laid in position over the cement."

A small round burnisher, which these gentlemen are directed to "keep thoroughly polished" (is there no limit?—no constitutional nerve paste?), is used to work the gold into the film of cement. Why not interject a direction about the oiling of the hand-piece? This cement suggestion as a starting base for the foil is so extremely puttering and unnecessary that it might better be omitted from a paper aiming to be scientific. It is this that I would characterize as an inconsequent something, for in years of practice equal to those of Dr. Rhein the necessity of its adoption has not even occurred to the writer. Its adoption by skilled operators is so improbable that one need be concerned only about its influence on the inexperienced, to whom the writer would say that it is far more expeditious, to put it mildly, having discreet undercuts in the floor and walls, to gather a mass of slightly annealed cylinders sufficient to cover the floor undercut liberally, and after locking the mass securely with hand-pressure to begin with the elected dynamic method.

In the time required for the hardening of the bit of cement to be of the conjectured aid every operator of ordinary ability with ordinary methods would have the filling well started, and many would have it one-third completed.

He sweetly, confidently, aye, benignly, next says, as if to some of second years, the cavity is now ready for the "strip of freshly annealed gold-foil" (tells these gentlemen how wide to cut it), which is malleted against the gold starting-point "cemented to the dental floor." If he has ever tried this on a flat cervical foundation devoid of undercut he has omitted to say how often it was found in the lap of the patient or on the floor.

At another point, in speaking of "a few moments for the small amount of cement to harden," he has the charming effrontery again, as if to a class of students, to direct his hearers to employ the time in cutting gold, yet fails to direct what the capable assistant, referred to in the next paragraph, is to do while the operator is cutting the gold.

He next says, "The filling is now brought in an even manner from the bottom upward, advancing no part of the gold beyond another part. This requisite evenness of surface is best accomplished by wiping the gold with a plugger, hammering away from *side to side*," "ironing" (my italics). How scientifically clear. "Hammering away [like a nailer] from side to side," and yet "advancing no part of the gold beyond another part."

This "requisite evenness" of the upbuilding is denied, for the reason that it is unavailing, inconsequent, and unnecessarily laborious to the operator and taxing to the patient.

While positive solidity throughout may be permissible, it is not a requisite for good service, and I would go so far as to aver distinctly that in many instances homogeneous impacting of gold is ill-advised and excessive, and furthermore that the even building up of a filling is inexpedient.

A broad foot-plugger is absolutely necessary to expeditiously create a hard regular surface to an increasing contour, and advance work with round pluggers by hand or otherwise in the undercuts, filling them liberally, will enable an operator to pack three-fourths of the approximal gold with the foot-plugger suggested, thereby accomplishing a saving of half the time required by the procedure outlined by Dr. Rhein. (Fig. 1.)

A homogeneous packing of the foil as a requisite is denied. Having the undercuts well packed by hand, the body or interior of the filling may be condensed but little better than could be well accomplished by hand if followed by perfectly condensed margins and surfaces, and such a filling will endure just as well as though perfectly condensed throughout.

On page 661 he offers another choice remark, where he states, "In fact, the best results are generally attained where the lower third or half of the filling is inserted at one sitting and the operation completed at a later time." In the language of the lurid litterateur, words fail me in attempting to characterize this puerility.

In the succeeding paragraph it is directed that the "matrices be removed occasionally," to see if the filling is loose. He gives his procedure a deserving negative in that very direction, and I truly believe with him that, with no undercut whatever in the foundation and none in the walls, the filling should be under suspicion at all times. Why conceive, much less exploit, a procedure so unassuring when positive methods have been in practice so long that many operators cannot remember when they last tested a filling for insecurity during its upbuilding.

He next says, "Care must be taken that all overhanging bits of gold should be removed and the polishing done without *in any way* [my italics] defacing or marring the enamel margins." This is an injunction impossible to adopt. Any means for the reduction of a rough surface of gold to a smoothness "resembling polished enamel" would affect the contiguous enamel, and it is impossible to reduce a filling to the directed degree of smoothness without "in any way defacing the enamel margins," no matter how much care is exercised. Care of the cervical enamel margins has been obligatory for so long that pointed cautionary reference to it in a paper to-day to an audience of past masters is not excessively deferential.

A strip or disk or file has some unavoidable effect on the contiguous cervical enamel, and may only be met by including these surfaces in the polishing process. The contiguous cervical enamel usually more or less disintegrated should be jealously conserved, and it is to be regretted that the essayist did not direct how it may be done without it "in any way defacing or marring the enamel margins."

Speaking of "best results," especially in distal cavities, it is not only very frequently humane, effective, and considerate to adopt a limited base of standard alloy (voicing my preference), and, as it may be made quite smooth by almost any wiping means, it offers practical immunity in the polishing to the glaze overlying the more or less disintegrated contiguous cervical enamel.

On page 662, in speaking of the finishing of this portion of the filling, he suggests that the day's work "stop at that point." Were this to be taken literally, one might inquire where the next "day's work" ceased. In the same paragraph he suggests (or audits) that the insertion of the filling be divided into several short sittings. The next bit of choice teaching is the frequent changing of the matrices to thinner ones, "until the very thinnest is used at the place mapped out for the marble-like contact points." These and the cement suggestion are of the same order. We are told that cement "sets in a few moments," that "best results are generally attained" by packing a part of the filling one day and completing it another, or *others* (dentistry as a pastime); that the matrix is to be removed occasionally (what joy!), to see if the filling is loose (what confidence!), and replaced with thinner ones, as if this were the only way or even an acceptable way of restoring contour. And yet, Dr. Rhein, in his reply to Dr. Wedelstaedt, says "that it becomes a difficult matter to reply to the same and preserve the temperate demeanor, which is only becoming to scientific discussion," implying thereby that he, Dr. Rhein, was and continues to be scientific.

The amount of time required to follow the technique urged by Dr. Rhein demands a clientele composed exclusively of the wealthy, enduring, and appreciative. I am free to urge that many patients have not these requisites sufficient to warrant, or the discrimination to appreciate, severely correct methods. Yielding to a maudlinity of theory, essayists so often seem unable to remember the realities of every-day practice, urging procedures that are decidedly inconsiderate of the consideration due the average patient.

By coupling vain theories with want of an ardor, a combination is created that is decidedly negative in many instances, and who can say how many teeth are lost or curtailed in usefulness because the discouraged patient, going to the other extreme, employs "Doctor Pusil," or neglects the dentist altogether. The suggestions by Dr. Rhein of two or more sittings offers no remedy in lessening the stress unless the patient possesses means; and, furthermore, the adjustment of the dam, clamps, etc., two or more times would indicate an amount of patience and endurance sufficient unto the stress of a restoration of any size at one sitting, except possibly in very rare instances. Perfect indication for two or more sittings for a single restoration are too rare to warrant the teaching that, "In fact, the best results are generally attained where the lower third or half is inserted at one sitting and the operation completed at a later time."

Ideal restorations are indicated perfectly only when the patient offers the usual combination of means, endurance, and discriminative appreciation, and in the degree that these favoring conditions are absent, a discreet operator lessens the amount of theory. With rare exceptions the capable operators throughout our profession do not and should not advise restorations that are taxing in every respect at all times, and what they do perform under the circumstances is in no wise culpable. The operator is not called upon, nor can he afford an extra hour of unremunerated finesse, any more than many patients are in a position to audit it financially or otherwise. Absolute homogeneity of impact in the average case is as unwise as unnecessary. Pursuing the subject to a greater attenuation, is it not reasonable to inquire what becomes of those cavities in which it is impossible to use a dynamic method throughout, wherein, as a result of the necessity of hand-pressure, a portion of the filling is not homogeneous to the degree pictured by Dr. Rhein? All operators of experience know that such a filling with condensed margins and surfaces is destined to last as long as one perfectly condensed throughout. In fact, we all audit imperfect condensation in many of the gold fillings we insert in daily practice, and if very fair condensation be audited at this point and at that because perfect condensation is denied, why not audit it for the major portion of the heart of the filling. Who can say but that it was this almost fanatical adherence to an ideal that contributed largely to the lamentable and untimely death of Dr. Marshall H. Webb, whose operations cannot be too highly extolled. I am personally indebted to him, and at the time of his death there were those in a memorial session of the Wisconsin State Dental Society who were moved to tears.

There can be no objection to ideally perfect condensation throughout, other than the unnecessary tax on the patient and operator, but as a matter of daily practice, if the comfort and well-being of the patient and operator are of any moment, I raise my voice against perfervid zeal as being ill-advised and intemperate.

To sum up Dr. Rhein's paper frankly, I am willing to believe that it is not his habit nor the habit of any good operator to mutilate a non-carious fissure, especially in a bicuspid, and, if that be the case, why mention a teaching so mischievous. Again I am willing to believe that he very rarely adopts the cement suggestion, and, if that be the case, why parade a teaching so puttering and unavailing?

As to his paper being lacking in deference, I hardly think that what I have said and implied needs modification.

## Figures and Tables

**Fig. 1. f1:**